# Measuring sustainment of prevention programs and initiatives: a study protocol

**DOI:** 10.1186/s13012-016-0467-6

**Published:** 2016-07-16

**Authors:** Lawrence A. Palinkas, Suzanne E. Spear, Sapna J. Mendon, Juan Villamar, Thomas Valente, Chi-Ping Chou, John Landsverk, Shepperd G. Kellam, C. Hendricks Brown

**Affiliations:** 1 School of Social Work, University of Southern California, Los Angeles, CA USA; 2 Department of Health Sciences, California State University, Northridge, CA USA; 3 Center for Prevention Implementation Methodology (Ce-PIM) for Drug Abuse and Sexual Risk Behaviors, Department of Psychiatry and Behavioral Sciences, Feinberg School of Medicine, Northwestern University, Chicago, IL USA; 4 Department of Preventive Medicine, Keck School of Medicine, University of Southern California, Los Angeles, CA USA; 5 Oregon Social Learning Center, Eugene, OR USA; 6 School of Public Health, Johns Hopkins University, Baltimore, MD USA

**Keywords:** Sustainment, Measures, Instruments, Implementation, Substance use, Suicide, Prevention

## Abstract

**Background:**

Sustaining prevention efforts directed at substance use and mental health problems is one of the greatest, yet least understood, challenges in the field of implementation science. A large knowledge gap exists regarding the meaning of the term “sustainment” and what factors predict or even measure sustainability of effective prevention programs and support systems.

**Methods/design:**

The U.S. Substance Abuse and Mental Health Services Administration (SAMHSA) supports a diverse portfolio of prevention and treatment grant programs that aim to improve population and individual level behavioral health. This study focuses on four SAMHSA prevention grant programs, two of which target substance abuse prevention at the state or single community level, one targets suicide prevention, and one targets prevention of aggressive/disruptive behavior in elementary schools. An examination of all four grant programs simultaneously provides an opportunity to determine what is meant by the term sustainment and identify and support both the *unique* requirements for improving sustainability for each program as well as for developing a *generalizable* framework comprised of core components of sustainment across diverse prevention approaches. Based on an analysis of qualitative and quantitative data of 10 grantees supported by these four programs, we will develop a flexible measurement system, with both general and specific components, that can bring precision to monitoring sustainment of infrastructure, activities, and outcomes for each prevention approach. We will then transform this system for use in evaluating and improving the likelihood of achieving prevention effort sustainment. To achieve these goals, we will (1) identify core components of sustainment of prevention programs and their support infrastructures; (2) design a measurement system for monitoring and providing feedback regarding sustainment within the four SAMHSA’s prevention-related grant programs; and (3) pilot test the predictability of this multilevel measurement system across these programs and the feasibility and acceptability of a measurement system to evaluate and improve the likelihood of sustainment.

**Discussion:**

This project is intended to improve sustainment of the supporting prevention infrastructure, activities, and outcomes that are funded by federal, state, community, and foundation sources.

## Background

Despite notable declines in the past 10 years in the USA, substance abuse among adolescents remains high; a third of tenth-graders and half of 12th graders have ever used an illicit drug, one in 15 high school seniors use marijuana daily and a quarter of seniors have had five or more drinks at one time in the last 2 weeks, a 10 % increase from 2011 to 2012 [1]. Adolescent drug abuse represents a substantial economic burden to society. Annually, drug abuse costs $600 billion [2]. Adolescent drug abuse also increases STD and HIV sex risk behavior with youths accounting for 39 % of all infections [3]. In terms of mental health, suicide among youths between the ages of 10 and 24 years is the second highest cause of death, and rates have been increasing [4]. Six percent of adolescent females and 2 % of males also attempt suicide each year, with 90 % of these youths having a diagnosable mental disorder [5]. Attempts are five to six times more common among those with an abuse/dependence disorder compared to those without such disorders [5, 6]. The high prevalence of psychiatric mood diagnoses and symptoms, combined with drug and alcohol use, place adolescents at a significantly higher risk of completing suicide [7, 8].

There are numerous evidence-based programs, practices, and initiatives designed to prevent substance abuse and suicide [9]. Specific prevention programs such as the Good Behavior Game (GBG) are cost effective in preventing drug and alcohol abuse and dependence disorders [10], criminal behaviors and antisocial personality disorder [11], suicide ideation and behavior [12], and HIV risk behavior [13]; this intervention is also highly cost effective and available for implementation [14, 15]. In contrast, programs focused on suicide are generally less definitive about their overall preventive effects. Other than our own work with GBG [12], few prevention programs have yet to demonstrate impact on both ideation and attempts, and no universal prevention program has demonstrated a significant reduction in suicide deaths [16].

Unfortunately, very few of these programs are routinely used, much less sustained when government funding comes to an end [9, 17]. The major research challenge we now face is not the lack of scientific knowledge about what works, but about how to integrate and maintain effective prevention programs, practices, policies, and principles in the institutions and communities charged with preventing drug abuse, sex risk behaviors, mental disorders, violence, and related outcomes.

Several models of research translation have been proposed over the years (see Damschroeder et al. [18] for a review). Many of these models consider sustainment to be the final stage of the process of implementation [19, 20], but the factors that predict sustainment are not well understood [21, 22]. In part, this may be attributed to a lack of consensus as to what constitutes sustainment and how to measure it. There are no uniform or agreed upon criteria for determining whether something has been sustained or not [23]. This may be due to the fact that what is to be sustained differs from one program to the next. For instance, with respect to the community coalitions supporting drug and suicide prevention activities, some definitions of sustainment focus on the coalition itself while others focus on the activities and impacts of the coalition [23]. Furthermore, with few exceptions [24–26], most studies reporting positive results have focused on earlier stages of implementation progress (exploration, adoption, routine use) and not on sustainment.

In addition to uncertainty as to how to define sustainment, there is a lack of consensus as to how to measure it. Chamberlain, Brown, and Saldana [27] developed the Stages of Implementation Completion (SIC), an eight-stage assessment tool developed as part of a large-scale randomized implementation trial. The stages range from engagement with the developers to practitioner competency and map onto three well-accepted phases of implementation—Pre-Implementation, Implementation, and Sustainability—the latter stage is currently only measured by a single stage 8 certification step. While the SIC is a measure of sustainment for a particular program, the ability of this instrument to measure intervention *sustainment* across different interventions has not yet been validated.

Another measure of sustainment is the Program Sustainability Assessment Tool [28], containing 40 items across eight sustainability domains, with five items per domain. The instrument developers reported high internal consistency reliability and some evidence of validity; however, the instrument has been used largely with evaluating chronic disease prevention programs and appeared to perform poorly with public health impact domains. They concluded that future research and evaluation work needs to be done to ascertain the validity and reliability of the instrument with different fields and types of interventions.

Federal agencies responsible for wide-scale delivery of prevention programs, including the Substance Abuse and Mental Health Services Administration (SAMHSA), routinely collect information from their grantees to monitor progress toward completion of goals and objectives. As part of their initial proposal for funding, all SAMHSA grantees are required to submit a plan for sustainment of the grantee’s activities once the funding has come to an end. SAMHSA programs currently rely on electronic data collection systems including the Transformation Accountability (TRAC) data collection system for SAMHSA’s Center for Mental Health Services (CMHS) programs, and the Coalition Online Management and Evaluation Tool (COMET) and the Performance Management Reporting Tool (PMRT) used by SAMHSA’s Center for Substance Abuse Prevention (CSAP). This information is used to provide feedback to grantees when there is evidence of failure to achieve goals and objectives. Currently, there is no empirical evidence that such feedback leads to an improvement in performance or increases the likelihood of sustainment.

While monitoring and feedback are recognized as important for prevention [15], much of the relevant science on feedback in health has involved improvement in clinical performance [29–32]. This includes clinical supervision and use of technology like electronic dashboards that monitor patient behavior and clinician activity [33–35]. Such feedback offers the clinician a better understanding of whether they are on course to achieve a successful outcome or need to alter their treatment strategy in order to improve the likelihood of a successful outcome. Similar measurement-based quality improvement (MBQI) strategies hold great promise for facilitating implementation and sustainment of evidence-based practices [36].

### Aims and objectives

SAMHSA supports a wide array of prevention grant programs targeting mental, emotional, and behavioral disorders including substance abuse, suicide, and antisocial behavior. Each of SAMHSA’s prevention initiatives has specific sets of goals and objectives, and each has different prevention approaches to be sustained once support from SAMHSA is no longer available. We will examine four SAMHSA prevention grant initiatives simultaneously to determine what is meant by the term sustainment in order to identify and support both the *unique* requirements for improving sustainment for each program as well as for developing a *generalizable* framework comprised of core components of sustainment across diverse prevention approaches. Based on an examination of grantees supported by these four programs, we will develop a flexible measurement system for sustainability, with both general and specific components that can bring precision to monitoring the structures and processes for sustaining each prevention approach. We will then transform this measurement system into a format that can be used to efficiently evaluate and improve the likelihood of achieving sustainment of any grantee’s prevention efforts, regardless of source of funding. To achieve these goals, we will:

1. Identify core components and their interrelationships across time for sustainment of prevention programs and their support infrastructures

Using ethnographic interviews, administrative data and network analysis of 10 grantees within four SAMHSA programs (Strategic Prevention Framework—State Initiative Grants, Sober Truth on Preventing Underage Drinking [STOP Act], Garrett Lee Smith Suicide Prevention Program, and Prevention Practices in Schools), we will identify relevant sustainment components, structures and functioning for agencies/organizations, community coalitions, and state prevention service systems that host prevention programs. This will enable us to determine whether or not a program has been sustained; what features of the intervention, grantee organization, external environment, implementation process, and individuals involved were critical to supporting that sustainment; and which of these features are common across all four programs.

2) Design a measurement system for monitoring and providing feedback regarding sustainment

Building on the results of aim 1, we will determine what additional information is necessary for SAMHSA to collect to determine the level and predictors of sustainment, the means for this data collection, and how this information can be organized into a brief scoring system for evaluating and improving the likelihood of sustainment.

3) Pilot test the predictability of the Sustainment Measurement System (SMS) and the feasibility and acceptability of this system to evaluate and improve sustainment likelihood

Using retrospective and prospective data from a larger sample of 100 grantee programs and analytic models based on advanced missing data procedures, we will examine the measurement system’s short-term predictability of sustainment in previous cohorts of these four SAMHSA programs. We will also evaluate the feasibility and acceptability for grantees of converting the information obtained from this measurement system into a format that can be used to provide feedback to grantees that will enable them to evaluate and improve their own progress toward sustainment.

## Methods/design

### Setting

Funded by SAMHSA’s Center for Substance Abuse Prevention (CSAP), the Strategic Prevention Framework—State Initiative Grant (SPF-SIG) Program has three goals: (1) prevent the onset and reduce the progression of substance abuse, (2) reduce substance abuse-related problems, and (3) build prevention capacity and infrastructure at the state, tribal, territory, and community levels through SAMHSA’s Strategic Prevention Framework (SPF) steps. These SPF steps require that grantees (a) assess their prevention needs based on epidemiological data; (b) build their prevention capacity; (c) develop a strategic plan; (d) implement effective community prevention programs, policies, and practices; and (e) evaluate their efforts for outcomes. Throughout all the five steps, grantees must address issues of sustainment and cultural competence [37]. Sustainment issues include the process through which a prevention system becomes a norm and is integrated into ongoing operations, particularly the statewide drug prevention block grants and prevention efforts at the local community level in that state. This infrastructure sustainment is vital to ensuring that prevention values and processes are firmly established, that partnerships are strengthened, and that financial and other resources are secured over the long term [37].

The Sober Truth on Preventing Underage Drinking (STOP) Act is a collaborative funded by the White House Office of National Drug Control Policy (ONDCP) and administered by CSAP. Eligible applicants are community coalitions with representation from 12 required sectors (Drug Free Communities Act of 1997, Public Law 105-20). This program works to achieve two goals: (1) establish and strengthen collaboration among communities, public and private non-profit agencies, and federal, state, local, and tribal governments to support the efforts of community coalitions working to prevent and reduce substance use among youths; and (2) reduce substance use among youths and, over time, reduce substance abuse among adults by addressing the factors in a community that increase the risk of substance abuse and promoting the factors that minimize the risk of substance abuse. STOP-Act-funded sites are eligible to receive additional mentoring grants to support new communities applying for STOP Act funding. Prominent sustainment issues include outcome sustainment, i.e., a continued reduction in substance use/abuse.

The Prevention Practices in Schools (PPS) Program is SAMHSA’s sole prevention grant that requires grantees to implement the Good Behavior Game (GBG), a classroom management strategy that involves helping children to learn how to work together through group contingent activities. Funded through SAMHSA’s Center for Mental Health Services (CMHS), GBG outcomes encompass the prevention goals of both CSAP—reduce substance abuse and smoking—and CMHS—reduce conduct disorder and suicidal ideation in youths. Eligible applicants for this program are local education agencies (school districts and tribal organizations); to date, 21 school districts and one tribal organization have been PPS grantees. Sustainment for PPS means that GBG would continue to be used in schools after SAMHSA funding has ended.

The Garrett Lee Smith Suicide (GLS) Prevention Program provides grant funding to states, tribes, and territories, as well as universities, state colleges, minority-serving institutions of higher learning, and community colleges. This program is community based and requires that funds be used by grantees for program development that directly address substance abuse and other behavioral health problems (e.g., depression), risks which are directly linked to suicide [7, 8, 38, 39]. GLS has six goals: (1) increased development and implementation of community-based suicide prevention programs; (2) training for recognition of at-risk behaviors; (3) improvement in access to and linkages with substance abuse and mental health services; (4) improvement and expansion of surveillance of suicide-related outcomes; (5) increased awareness of suicide as a public health problem; and (6) development and implementation of strategies for reducing stigma associated with services for mental health and suicide prevention activities. The most common prevention approaches across grantee communities and organizations have been gatekeeper training and screening programs to identify youths at risk [8]. SAMHSA has also emphasized the need for community collaborations and asks all sites to evaluate how well community coalitions have been developed through GLS. Sustainment issues emphasize continued coalition support and delivery of programs that positively affect risk and protective factors for suicide as well as monitoring of outcomes [8].

As illustrated in Table 1 below, the four programs provide shared as well as unique aspects of the problems of monitoring public support for the phases of implementation. The four programs were selected by SAMHSA for investigation because they represent the agency’s new strategic vision to align programs with the delivery of integrated behavioral health services as part of the Affordable Care Act of 2011. Preventive interventions range from a single evidence-based program (GBG) to combined strategies (SPF-SIG and STOP Act) to best available program where evidence is still being accumulated from trials (GLS). Further, all of these four SAMHSA grant programs share the problem of assessing and attending to the building and maintaining of public support for each stage of implementation including sustainment. They allow the development of a general method with specific as well as shared characteristics across different programs.

**Table 1:** Comparison of SAMHSA study programs

	Strategic Prevention Framework—State Incentive Grants (SPF-SIG)	Sober Truth on Preventing Underage Drinking (STOP Act)	Garrett Lee Smith Suicide Prevention (GLS)	Prevention Practices in Schools (PPS)
SAMHSA program	CSAP	CSAP	CMHS	CMHS
No. of grantees	60 block grants 35 SPF-SIG cohorts 4-5 46 State Prevention Enhancement	608 continuations 60 new grantees 22 mentoring continuation grantees 6 new mentoring grantees	27 states and 26 tribes currently funded	21 active
Assessment of progress toward achieving goals/aims	Web Block Grant Application System (BGAS) Performance Management Reporting Tool (PMRT)	Coalition Online Management and Evaluation Tool (COMET)	Cross-site evaluation Transformation Accountability (TRAC) data collection system Local performance assessment	Transformation Accountability (TRAC) data collection system, monthly phone calls with GPO, and annual reports
Frequency of evaluation	Annual review of GRPA and NOMs data and progress reports quarterly	Review of COMET reports twice a year Site visits every 3 and 5 years	Quarterly and annually	Monthly, quarterly, and annually

### Conceptual framework

**Fig. 1:**
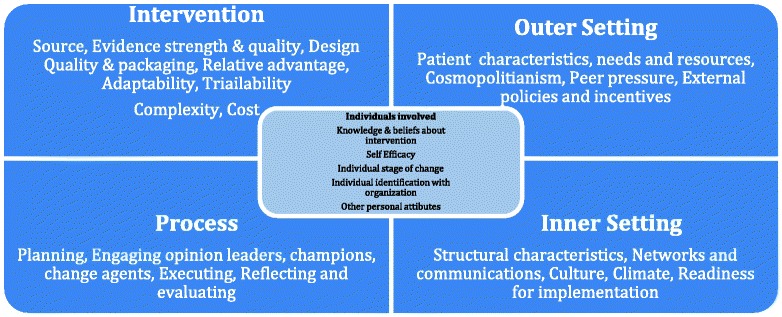
Consolidated Framework for Implementation Research (CFIR)

The Consolidated Framework for Implementation Research (CFIR) [18] offers an overarching typology for implementation research and comprises five major domains: the intervention, inner and outer setting in which it is implemented, the individuals involved in implementation, and the process by which implementation is accomplished. An illustration of this framework and the components of each domain is provided in Fig. 1. Elements of sustainment can be measured across all five dimensions, e.g., for intervention, whether a particular program continues after funding; for process, whether a strategic prevention framework is used; for inner setting, whether a school system maintains the infrastructure to support a program; for outer setting, whether a community coalition continues to commit to a prevention plan; and for individuals, whether a superintendent maintains support. By including a dynamic perspective where changes in each of these five settings occur, this framework also enables us to test what factors are predictors of sustainment.

Although many factors influence the implementation of evidence-based practices (EBPs), researchers have consistently found that interpersonal contacts within and between organizations and communities are important influences on the adoption of new behaviors. Based on Diffusion of Innovations Theory [40] and Social Learning Theory [41], studies and meta-analyses have shown that both the influence of trusted others in one’s personal network and having access and exposure to external information are important influences on rates of adoption and implementation of innovative practices [42–44]. Social networks have also been viewed as an important characteristic of community coalitions [45–47]. Feinberg and colleagues [48] found that network cohesion to be positively associated and network centralization to be negatively associated with community readiness to engage in the Communities That Care community-based prevention coalition. Bess and colleagues [45] found that initial coalition participation in a youth violence prevention program was associated with a pre-existing network of interorganizational relations. Hence, we will pay particular attention to the social networks of organizations implementing SAMHSA-funded programs.

### Overview of study design

The project will proceed in three phases. Phase 1 involves ethnographic fieldwork, guided by the CFIR framework in 10 selected sites across the four programs and the analysis of archival administrative data routinely collected by SAMHSA to evaluate grantee performance. Our aim in this phase is to characterize what sustainment means across programs and extract shared and unique characteristics. Phase 2 involves the creation of the new measurement system incorporating data already being routinely collected along with additional data identified in Phase 1. Phase 3 involves the collection and analysis of data from SAMHSA grantees to validate the system as a means of monitoring progress toward sustainment of coalition process and products.

### Phase 1

#### Participants

In collaboration with SAMHSA’s CMHS and CSAP associate directors and senior program staff, we identified two to three grantees within each of the four SAMHSA-funded programs and solicited their participation in the proposed project. These 10 sites include two PPS grantees currently implementing the Good Behavior Game, three SPF-SIG grantees, two STOP Act grantees, and three GLS grantees. These sites were purposefully sampled [49] to reflect diversity with respect to race/ethnicity, geography, quality of evidence supporting funded activities (i.e., the extent to which they are evidence-based or “evidence-informed”) and perceived level of success in achieving sustainment of program activities, infrastructure, or outcomes.

During a 2–3-day visit at each site, investigators will conduct individual semi-structured interviews with the grantee principal investigator, the project coordinator, and a minimum of four key informants representing coalition or community partners purposefully sampled on the basis of the site PI’s assessment of level of engagement in the project (two least engaged and two most engaged). In addition to these detailed interviews, all members of a coalition will also be invited to complete a brief questionnaire containing questions relating to types of interactions among coalition members and their social network relations.

#### Data collection

Participating phase I SAMHSA grantees will provide project investigators with a copy of the original grant proposal, progress reports to SAMHSA, minutes of meetings with SAMHSA representatives, and any reports or publications disseminated outside SAMHSA. The hour-long interviews will be conducted with the use of an interview guide and comprised of three parts: (1) a series of semi-structured questions relating to experience with implementing and sustaining the program; (2) a free list exercise [50]; and (3) a template [51] of CFIR domains and components. In particular, we are interested in knowing the following: (1) what, if anything, they wanted to sustain; (2) how they perceive whether it was, in fact, sustained or not; and (3) their ranking of key determinants of sustainment.

All coalition members identified by the site PI, program coordinator, and key informants will be asked to complete a brief web-based survey that includes the full sampling frame of the site coalition thought to engage in the SAMHSA-funded initiative. They will then be asked how long they have known these individuals (in years) and to indicate whether (1) they had worked with each other member in the past year on any issue; (2) worked together in the past year on SAMHSA-funded initiative, program or practice issues; and, if yes, then (3) what types of collaboration each relationship involved (prompting for six areas, including advocacy and policy work, information sharing, program delivery, resource sharing, service delivery, or training/education; and (4) which of the defined members they considered a leader or innovator in the SAMHSA-funded initiative, program, or practice.

#### Data quality and management

All interviews will be digitally recorded and transcribed. Interviewers will then compare transcripts with digital records to insure accuracy of transcription. All field notes, interview transcripts, and interviewer notes summarizing interview and focus group experience will be entered into Dedoose [52]. A data accounting and back-up system will be instituted to keep track of, and facilitate access to, all electronic and hard-copy data.

To insure credibility of findings and enhance the validity and reliability of data collected, all interviews will be reviewed by at least two members of the research team. As described in detail below, consensus on coding and coding procedures and modifications to coding books will occur through regular team meetings. When possible, study results will be presented to informants and other study participants, enabling them to provide comment of results and suggest modifications or additional avenues of investigation. An audit trail of data collected as well as memos and minutes of team meetings, indicating time, place, persons providing information, and persons collecting or analyzing information, will be kept throughout the study.

#### Qualitative data analysis

Three types of qualitative analysis will be used with the data collected during this phase. First, using a methodology of “Coding Consensus, Co-occurrence, and Comparison” developed for prevention research qualitative analysis [53], field notes and interview transcripts will be analyzed in the following manner. Each investigator will review this material and prepare short descriptive statements or “memos” to document initial impressions of topics and themes and their relationships and to define the boundaries of specific codes (i.e., the inclusion and exclusion criteria for assigning a specific code) [54]. Segments of text in field notes and interview transcripts will be assigned codes based on a priori (i.e., from the interview guide) or emergent themes (also known as open coding [55]). Lists of codes developed by each investigator will be matched and integrated into a single codebook. Each text will be independently coded by at least two investigators. Disagreements in assignment or description of codes will be resolved through discussion between investigators and enhanced definition of codes. The final list of codes or codebook will consist of a numbered list of themes, issues, accounts of behaviors, and opinions that relate to program structure, function, development, and sustainment. With the final coding structure, two investigators will separately review transcripts to determine level of agreement in the codes applied. A level of agreement in the codes applied ranging from 66 to 97 % depending on level of coding (general, intermediate, specific) indicates good reliability in qualitative research [56]. Based on these codes, Dedoose will be used to generate a series of categories arranged in a treelike structure connecting text segments grouped into separate categories of codes or “nodes.” These nodes and trees will be used to further the process of axial or pattern coding [55] to examine the association between different a priori and emergent categories. Through the process of constantly comparing these categories with each other, the different categories will be further condensed into broad themes using a format that places program formation, structure, and functioning within the framework of the site’s organizational and system context.

Second, the free lists of characteristics of and requirements for sustainment will be tabulated by counting the number of respondents who mentioned each item and then ordering in terms of frequency of responses. Multidimensional scaling analysis [50] will then be used to identify common and unique characteristics in each of the four SAMHSA-funded programs believed to be associated with sustainment.

Finally, a matrix of sustainment characteristics and requirements will be developed for each of the four SAMHSA-funded programs with a list of the CFIR domains and components on one dimension and the classification of sustainment (e.g., infrastructure, intervention, and outcomes) on the other dimension. Both the organization and content of subgroup matrices will be compared to identify features of coalitions that influence the likelihood of achieving sustainment on each dimension that are specific to particular SAMHSA programs and features common to all four programs [51]. Comparison of these matrices will also enable project investigators to compare the project director, coordinator, and coalition member perspectives elicited during extended semi-structured interviews. The project investigators will then select a set of common and specific elements to be incorporated into the design of a heuristic model of sustainment.

#### Social network analysis

The matrix of ties used to analyze advice networks will be constructed from data collected from the web-based survey, supplemented by data collected during the qualitative interviews [57]. The social network analysis will proceed in three stages: network visualization, structural analysis, and statistical analysis of outcomes. The network visualization will be accomplished using NetDraw 2.090. The spring embedder routine will be used to generate the network visualizations [58]. Structural analyses will then be conducted on these network data using Ucinet for Windows, Version 6 [59]. Several network level measures of structure will be assessed, including total number of ties, network size, density (the number of reported links divided by the maximum number of possible links), average distance between nodes, and the number of components (i.e., unique sub-networks). To assess status and interconnectivity within the network, we will calculate degree centrality for incoming ties (being nominated by alters) and outgoing ties (nominating alters). In-degree and out-degree centrality scores assess the relative status of a given node. We will also examine several other measures of network status, including between-ness, closeness, and eigenvector centrality. Eigenvector centrality also allows one to examine in- relative to out-ties, but in- and out-degree centrality correspond directly to counts of nominations by and toward an actor, and as such have a straight-forward substantive interpretation, which eigenvectors lack. Homophily (i.e., likeness between individuals in a network based on specified criteria) data will be assessed on two key variables of interest, the SAMHSA program funding the grantee and sustainment (sustained, not-sustained). Homophily scores can be regarded as the proportion of individuals in a person’s network who share a characteristic with that individual.

### Phase 2

During this phase, we will identify data already being collected by SAMHSA corresponding to the relevant CFIR domains and components identified in phase 1. These data will be found in the Transformation Accountability (TRAC) data collection system for the GLS and PPS grantees, the Coalition Online Management and Evaluation Tool (COMET) for the STOP Act grantees, and the Performance Management Reporting Tool (PMRT) for the SPF-SIG grantees. This identification will include sustainment components that are collected across all four programs and components that are unique to each program.

Second, we will identify data relevant to sustainment not routinely collected by SAMHSA. This will be accomplished by comparing the matrix for relevant data elements identified in phase 1 with the list of common and unique sustainment data elements identified in the first activity of phase 2.

Third, we will design the Sustainment Measurement System (SMS), which integrates data currently being collected as part of existing SAMHSA data collection and reporting systems, and data that can be used to assess progress toward and likelihood of sustainment of project infrastructure, process (i.e., interventions and activities supported by the infrastructure), and outcomes. The SMS will be similar to the Stages of Implementation Completion (SIC) measure described above but involve ordinal scales with three or four categories so that sophisticated Item Response Theory (IRT) analyses can be conducted (see “Data analysis” section below). Like the SIC, it will also have separate sections pertaining to different roles in implementation, e.g., grantee administration, community coalition, and program home-site coordinator. In this instance, the tool will consist of all the elements identified in phase 1 as being relevant (i.e., a potential predictor or requirement) to sustainment of project infrastructure, process, and outcomes. The tool will also include the respondent’s assessment of whether or not sustainment has been achieved with respect to each of these categories, weighted on the basis of priority assigned by respondent to each category. Respondents will be asked to indicate what sustainment components are present and when key benchmarks of process were achieved.

A prototype measurement system will be developed that includes (1) data requesting (e.g., by whom, when); (2) data integration (e.g., with SAMHSA and additional data); (3) analytics (e.g., index to predict sustainment); and (4) visualization (e.g., profile to identify strengths and weaknesses). This will be developed in collaboration with SAMHSA as well as grantees to ensure utility and usability.

### Phase 3

#### Participants

All site directors and collaborators or coalition representatives of all of the current grantees funded by the four SAMHSA programs will be invited to participate in this phase of the project. This will include all 21 grantees funded by Prevention Practices in Schools, 53 states and tribes funded by the Garrett Lee Smith Suicide Prevention Program, 35 SPF-SIG grantees, and 120 STOP Act grantees (estimated total site *n* = 230, see Table 1). Eligibility for study participation includes the following: (1) project is currently being funded or funded within the past 2 years by one of the four SAMHSA programs (PPS, SPF-SIG, STOP Act, or GLS), (2) project has submitted an annual progress report to SAMHSA for a minimum of 1 year, and (3) project is expected to end funding within this R34 grant period. There are 188 grantees close to the sustainability stage; we anticipate 140 responses in Years 02 and 100 at the 6-month and 12-month follow-up.

#### Data collection

Each eligible SAMHSA-funded grantee will be invited to complete a web-based survey during Years 2 and 3. The PI or program coordinator will be asked to provide permission to access data already submitted to SAMHSA as part of its mandatory progress reporting system or procedures. These data will be limited to only that information relevant to the SMS. The survey will consist of a series of questions relating to SMS elements that are not currently or have not been previously collected by SAMHSA. These would include information relating to social networks of project coalitions or collaborators and other domains and components of the CFIR found in Phase 2 to be potentially relevant to the sustainment of program structure, process, and outcomes.

#### Data analysis

Using Mplus 7.11 [60] and the *R* statistical package, a series of statistical analyses will be conducted to assess which factors appear to be strongest determinants of sustainment, taking account of the time to event (i.e., different lengths of follow-up since program termination). Since sustainment involves multiple subdimensions and shared as well as unique components, we will first examine one- and two-dimensional models of the latent structure of sustainment items, which are measures on three- to four-point scales in order to conduct item response theory (IRT) analyses. One-dimensional latent factor structure models of all items across infrastructure, process, interventions, and outcomes will be attempted first, but we anticipate that more complex models will be required. Specifically, we propose using two types of bifactor models for IRT analyses [61] since these decompose each item into a common factor (i.e., shared sustainment) and a second specific factor relevant to that particular subdimension or SAMHSA program. In bifactor models, the common factor score for an item can be used to assess the level of sustainment shared across all SAMHSA programs, while the specific factor characterizes that second dimension. For our first bifactor model, we would characterize how an item’s specific loading involves that item’s position in the CFIR framework (e.g., inner, outer, process, intervention, person). The second bifactor model would characterize each specific factor loading as pertaining to that particular SAMHSA program and therefore can be used to rank grantees within each grant program. A third analysis will integrate these into a comprehensive model that includes covariates and time.

We will then use the predictors from our Sustainability Measurement System in a latent variable model with the outcome being the latent common sustainment score from the bifactor model described above. Continuous measures, including the time that the grant was originally funded and categorical measures such as which of the four grant programs provided funding will be used to assess developmental as well as unique versus specific predictors in these analyses. Items that are specific to particular programs can be treated as informative indicators themselves or “missing at random” and thereby all measures across all grantees can be included in analyses.

## Discussion

The project is innovative in three specific respects. First, unlike other projects that focus on only one practice or program, we will be simultaneously examining sustainment of infrastructure, activities, and outcomes in four different sets of SAMHSA-funded programs. This will give us a rare opportunity to identify a set of common elements of sustainment that can be used to generate a model and set of testable hypotheses that apply to a broad array of drug abuse/mental disorder/suicide prevention programs, practices, and initiatives, regardless of objectives, outcomes, and infrastructure to achieve these outcomes. Second, we are developing a measure of sustainment that can be used validly and reliably across this broad array of programs, practices, and initiatives with varying levels of evidence to support their effectiveness. This will enable us to determine whether the extent to which a program or practice is evidence-based or evidence-informed determines whether it can be sustained [19–21]. Third, we will tailor this measure so that it can be used to monitor progress toward sustainment and provide feedback to stakeholders as to how to increase the likelihood of sustainment. This measurement system will thus have use as a tool for program management as well as research purposes. Although this project targets programs funded by SAMHSA, the work should have general applicability across diverse federal, statewide, and local prevention implementation initiatives.

## Abbreviations

CFIR, Consolidated Framework for Implementation Research; CMHS, Center for Mental Health Services; CSAP, Center for Substance Abuse Prevention; SAMHSA, Substance Abuse and Mental Health Services Administration; SIC, Stages of Implementation Completion
